# Pathophysiological analyses of cortical malformation using gyrencephalic mammals

**DOI:** 10.1038/srep15370

**Published:** 2015-10-20

**Authors:** Kosuke Masuda, Tomohisa Toda, Yohei Shinmyo, Haruka Ebisu, Yoshio Hoshiba, Mayu Wakimoto, Yoshie Ichikawa, Hiroshi Kawasaki

**Affiliations:** 1 Department of Biophysical Genetics, Graduate School of Medical Sciences, Kanazawa University, Ishikawa 920-8640, Japan; 2 Brain/Liver Interface Medicine Research Center, Kanazawa University, Ishikawa 920-8640, Japan; 3 Department of Neurology, Graduate School of Medicine, The University of Tokyo, Tokyo 113-0033, Japan

## Abstract

One of the most prominent features of the cerebral cortex of higher mammals is the presence of gyri. Because malformations of the cortical gyri are associated with severe disability in brain function, the mechanisms underlying malformations of the cortical gyri have been of great interest. Combining gyrencephalic carnivore ferrets and genetic manipulations using *in utero* electroporation, here we successfully recapitulated the cortical phenotypes of thanatophoric dysplasia (TD) by expressing fibroblast growth factor 8 in the ferret cerebral cortex. Strikingly, in contrast to TD mice, our TD ferret model showed not only megalencephaly but also polymicrogyria. We further uncovered that outer radial glial cells (oRGs) and intermediate progenitor cells (IPs) were markedly increased. Because it has been proposed that increased oRGs and/or IPs resulted in the appearance of cortical gyri during evolution, it seemed possible that increased oRGs and IPs underlie the pathogenesis of polymicrogyria. Our findings should help shed light on the molecular mechanisms underlying the formation and malformation of cortical gyri in higher mammals.

The cerebral cortex is crucial for higher brain functions and is especially developed in higher mammals including humans. One of the prominent features of the cerebral cortex of higher mammals is the presence of gyri. Humans, monkeys and ferrets have gyrencephalic brains (i.e. brains with a folded cerebral cortex), while the brains of rodents are often lissencephalic (i.e. lacking cortical folds). Because malformations of the cortical gyri during development are associated with severe disability in brain function and diseases such as lissencephaly, polymicrogyria, epilepsy, schizophrenia and autism^1^,^2^,^3^,^4^,^5^,^6^,^7^, the mechanisms underlying the development and malformation of the cortical gyri have been of great interest.

Thanatophoric dysplasia (TD, see also Supplementary Table) is a relatively common skeletal dysplasia in which the cerebral cortex displays a unique and complex malformation^8^,^9^. The cortical malformation of TD is characterized by a combination of abnormalities including polymicrogyria, enlargement of the cerebral cortex (megalencephaly), subarachnoid heterotopia and subependymal heterotopia^8^,^9^. TD is caused by mutations of the *fibroblast growth factor receptor 3* (*FGFR3*) gene^10^,^11^,^12^,^13^,^14^,^15^ which lead to constitutive activation of FGFR3 tyrosine kinase activity^16^,^17^. Although it has been established that the activation of FGF receptors affects cell proliferation and differentiation during development, the link between constitutive activation of FGFR3 and the cortical malformation is still largely unclear^8^. This is at least partially because embryonic brains of patients with cortical malformations are often hard to obtain. Therefore, appropriate animal models would be extremely important for understanding the pathophysiology of cortical malformations.

Recent pioneering studies demonstrated that a mouse model of TD, which has the same activating mutation in the mouse *FGFR3* gene as that which occurs in human TD, showed cortical malformations^18^,^19^. Megalencephaly was found to be a prominent phenotype of mouse TD, like human TD. Interestingly, however, the cortex in mouse TD did not form abnormal sulci and gyri, although human TD patients have abnormalities in the cortical gyri^18^,^19^. This result raised the possibility that mouse TD did not recapitulate the entire pathophysiology of human TD, presumably because normal mice do not have gyri and sulci on the cerebral cortex. Therefore, to examine the mechanisms of the formation and malformation of cortical gyri in higher mammals, we have utilized gyrencephalic carnivore ferrets, which have been widely used for neuroscientific research^20^,^21^,^22^,^23^. Furthermore, to manipulate gene expression in the ferret cerebral cortex, we recently established a rapid and efficient gene manipulation technique for ferrets using *in utero* electroporation^24^,^25^. Using this technique, here we successfully produced the cortical phenotypes of TD by expressing fibroblast growth factor 8 (FGF8) in the ferret cerebral cortex. Strikingly, in contrast to TD mice, TD ferrets showed not only megalencephaly but also abnormalities in the cortical gyri such as polymicrogyria and subependymal heterotopia. Therefore, we were able to investigate the pathophysiology of TD using embryonic and neonatal TD ferrets. Our findings should help shed light on the molecular mechanisms underlying the formation and malformation of the cortical gyrus in higher mammals.

## Results

### FGF8 induces cortical malformation in the ferret cerebral cortex

To make a TD model animal, we expressed FGF8 in the cerebral cortex of gyrencephalic ferrets because FGFR3, which is responsible for TD, is preferentially activated by FGF8^26^,^27^. We performed *in utero* electroporation to co-express mouse FGF8 and GFP in the cerebral cortex of ferret embryos at E33 as described previously^24^,^25^. To detect the distribution patterns of ectopically expressed mouse *FGF8* mRNA selectively, we performed *in situ* hybridization with a mouse FGF8 probe using the transfected ferret cerebral cortex. As expected, mouse *FGF8* mRNA was indeed expressed in GFP-positive cells (Supplementary Fig. S1), suggesting that the distribution patterns of GFP-positive cells represent those of ectopically expressed *FGF8* mRNA.

**Figure 1:**
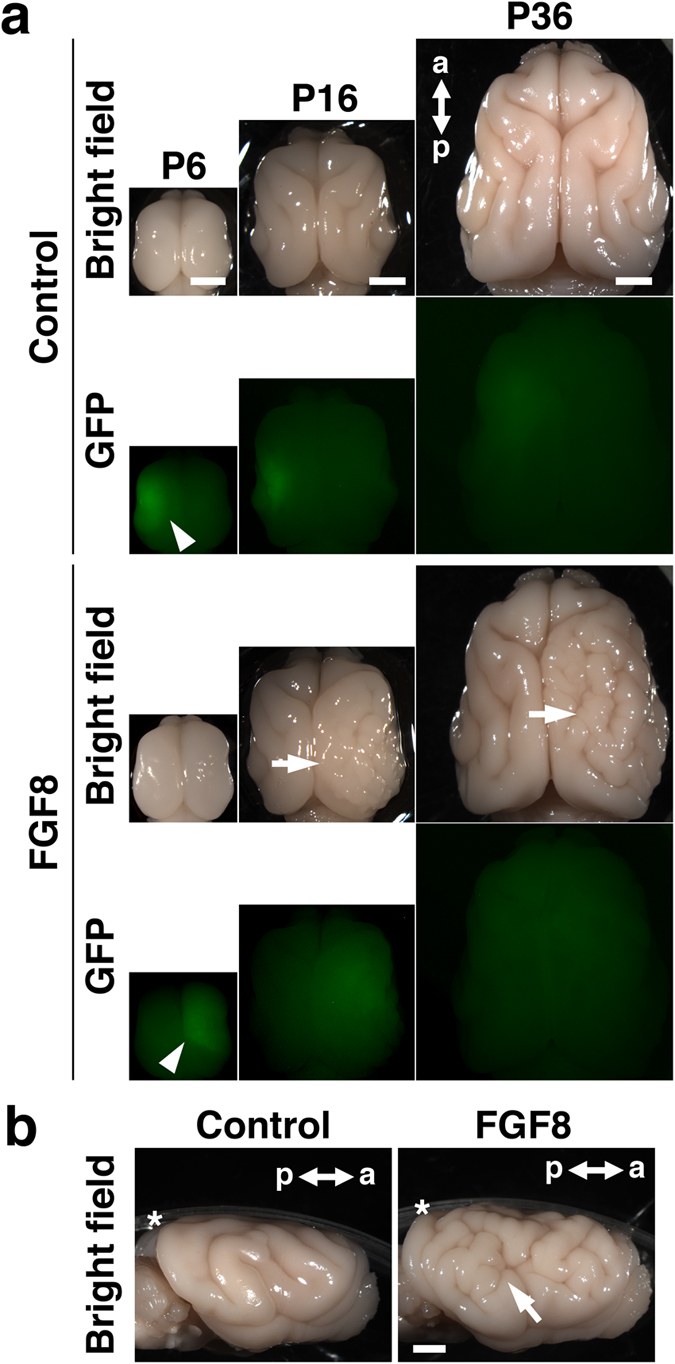
FGF8 induces polymicrogyria and megalencephaly in the developing ferret brain. (**a**) Dorsal views of the developing ferret brain. GFP or GFP plus FGF8 were expressed in one side of the brain at E33 using *in utero* electroporation, and the brain was prepared at the indicated time points. Electroporated areas showed GFP fluorescence (arrowheads). FGF8 induced polymicrogyria and megalencephaly at P16 and P36 (arrows). (**b**) Lateral views of the ferret brain at P36. The arrow indicates polymicrogyria. Asterisks indicate plastic dishes. a, anterior; p, posterior. Scale bars = 4 mm.

After electroporation was performed at E33, brain samples were obtained at P6, when cortical gyri have not yet been formed; at P16, when they are starting to form; and at P36, when they have been formed^28^. In control ferrets, the cortical surface was smooth, and the cortical gyrus was not evident at P6 (Fig. 1a, control). Consistent with previous studies^28^, cortical gyri were visible at P16, and became more prominent at P36 (Fig. 1a, control). Cortical sulci such as the coronal sulcus and the suprasylvian sulcus were clearly visible. Importantly, there were no apparent differences in the patterns of cortical gyri between the GFP-electroporated side and the other side in control ferrets (Fig. 1a, control), suggesting that genetic manipulation using *in utero* electroporation does not have obvious adverse effects on gyrification.

**Figure 2:**
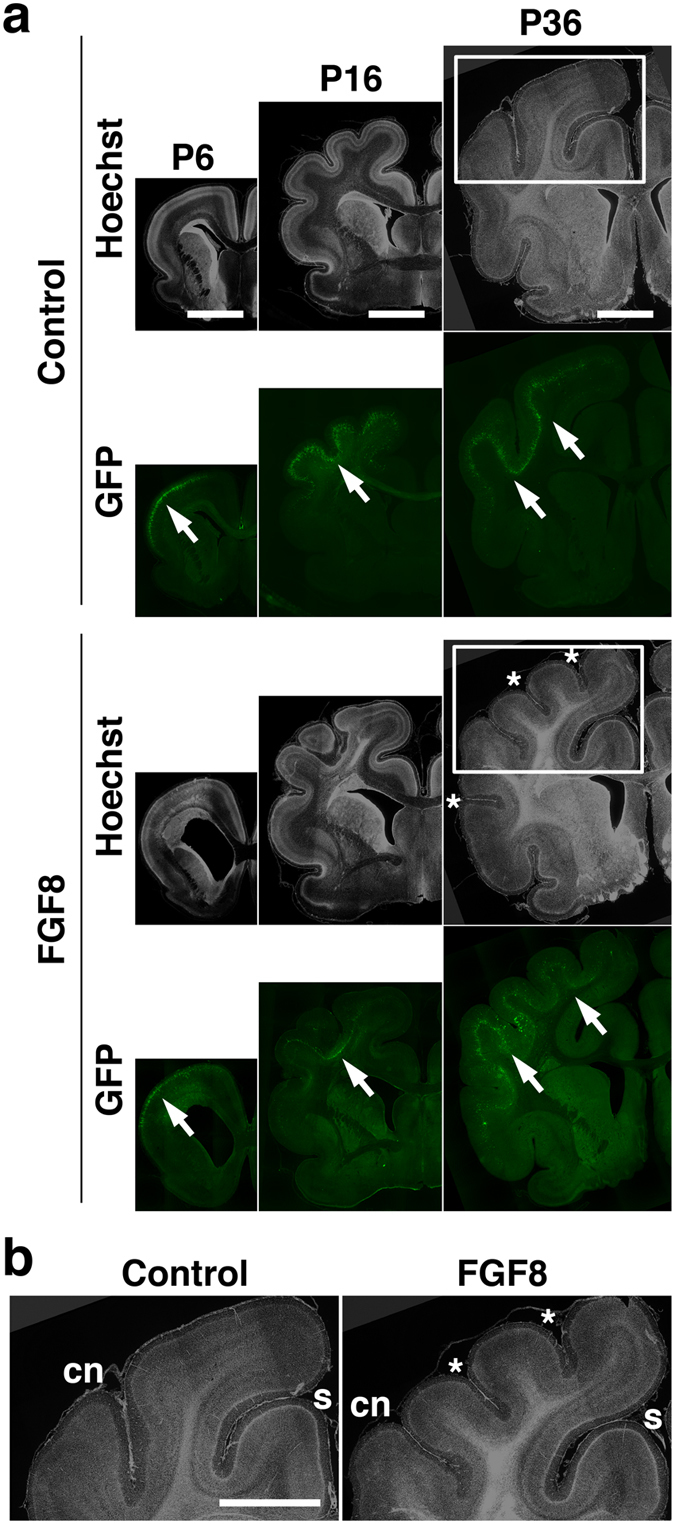
Histological examination of the effects of FGF8 in the developing ferret brain. (**a**) GFP and FGF8 were expressed in the ferret cerebral cortex at E33 using *in utero* electroporation, and the brain was prepared at the indicated time points. Coronal sections were prepared and stained with Hoechst 33342. Additional sulci are clearly visible at P36 (asterisks). Electroporated areas showed GFP fluorescence (arrows). Scale bars = 6 mm. (**b**) Magnified images in the white boxes in (**a**). Newly formed sulci are indicated by asterisks. s, splenial sulcus; cn, coronal sulcus. Scale bar = 6 mm.

Interestingly, we found that the FGF8-transfected cortex had more complicated gyrus patterns than the GFP-transfected control cortex at P16 (Fig. 1a, arrow). While sulci and gyri that existed on the control brain were similarly distributed on the FGF8-transfected cortex, a number of undulating folds were added where FGF8 was expressed. Newly added folds became more prominent at P36 (Fig. 1a,b, arrows). These results suggest that the FGF8-transfected cortex shows polymicrogyria, which is one of the most common phenotypes of TD^8^,^9^. In addition to the patterns of cortical folding, the brain of the FGF8-electroporated side was larger than that of the other side (Fig. 1a). Our quantification showed that the FGF8-transfected hemisphere was significantly larger than the GFP-transfected control hemisphere at P6 (control, 1.023 ± 0.029; FGF8, 1.245 ± 0.063; Student’s *t*-test; p < 0.05), P16 (control, 0.967 ± 0.015; FGF8, 1.104 ± 0.028; Student’s *t*-test; p < 0.01) and P36 (control, 0.981 ± 0.014; FGF8, 1.327 ± 0.091; Welch’s *t*-test; p < 0.05) (Supplementary Fig. S2a–c). This is consistent with the fact that megalencephaly is a common phenotype of TD^8^. We also measured the size of the lateral ventricle and found that the lateral ventricle was significantly larger in the FGF8-transfected hemisphere than in the GFP-transfected control hemisphere at P6 (control, 1.02 ± 0.17; FGF8, 3.63 ± 0.61; Student’s *t*-test; p < 0.01), P16 (control, 0.83 ± 0.20; FGF8, 2.82 ± 0.88; Student’s *t*-test; p < 0.05) and P36 (control, 1.02 ± 0.42; FGF8, 4.37 ± 0.51; Student’s *t*-test; p < 0.01) (Fig. 2a and Supplementary Fig. S2d–f). The enlargement of the lateral ventricle could contribute to megalencephaly.

Interestingly, although FGF8-electroporated ferrets exhibited polymicrogyria, it was reported that polymicrogyria was not found in TD mice^18^,^19^. This could be because ferrets have gyrencephalic brains, and therefore ferrets seemed to be more appropriate for investigating the mechanisms of cortical malformation than mice. These findings suggest that FGF8-transfected ferrets are useful for examining the pathophysiology of TD in the brain (hereafter FGF8-transfected ferrets are referred to as TD ferrets).

### Characterization of cortical folding in TD ferrets

We quantified the effect of FGF8 on cortical folding using coronal sections. Coronal sections containing the anterior part of the lateral ventricle were used for further analyses. Consistent with our macroscopic observations (Fig. 1), coronal sections showed increased cortical folds in the FGF8-electroporated cortex compared with the GFP-electroporated control cortex at P36 (Fig. 2, asterisks).

**Figure 3:**
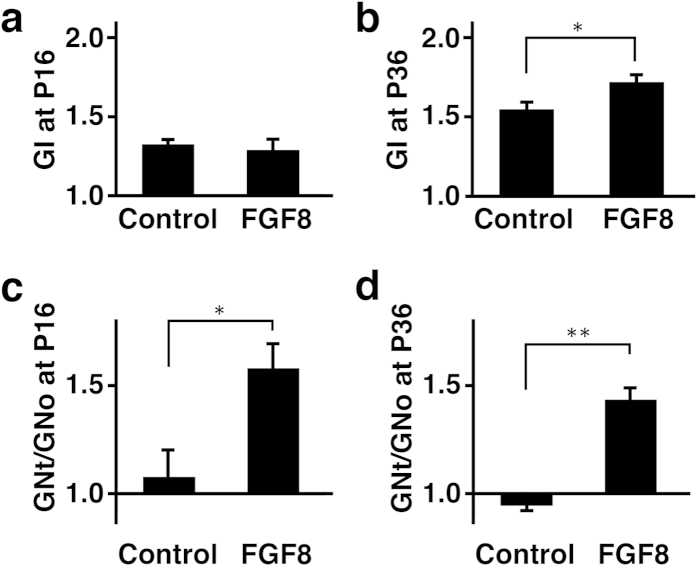
Quantification of the effects of FGF8 on gyrification. GFP and FGF8 were expressed in the ferret cerebral cortex at E33 using *in utero* electroporation, and the brain was prepared at the indicated time points. Coronal sections containing the anterior part of the lateral ventricle were used for quantification. (**a**,**b**) The GI values were measured using the electroporated side of the brain. The GI values were significantly larger in the FGF8-transfected brain at P36 (**b**). (**c**,**d**) The GN values were measured, and the GNt/GNo values were calculated. The GNt/GNo values were significantly larger in the FGF8-transfected brain at P16 and P36. Bars represent mean ± SD. *p < 0.05; **p < 0.01. n=3 for each condition.

We measured the length of the complete contour (i.e. the pial surface) and that of the outer contour of the brain using coronal sections, and calculated the gyrification index (GI), which is the ratio of the length of the complete contour to that of the outer contour (See the Methods section and Supplementary Fig. S3 for details)^29^. The GI values would be 1 if there were no cortical folds, and would become larger numbers if cortical folds were formed. Indeed, the GI values were almost 1 at P6, when cortical folds were not evident, and became larger thereafter in control ferrets as cortical gyri appeared (P16, 1.32 ± 0.03; P36, 1.55 ± 0.04; Student’s *t*-test, p < 0.01) (Fig. 3a,b). Consistent with our macroscopic observations (Fig. 1), we found that the GI values were significantly larger in the TD ferret brain than in the control brain at P36 (control, 1.55 ± 0.04; TD, 1.72 ± 0.04; Student’s *t*-test, p < 0.05) (Fig. 3b). These results suggest that FGF8 induces ectopic cortical folds. Although our macroscopic observations found polymicrogyria as early as P16 (Fig. 1), the GI values did not show significant differences between TD and control ferrets at P16 (control, 1.32 ± 0.03; TD, 1.28 ± 0.07; Student’s *t*-test, p > 0.05) (Fig. 3a). This discrepancy was likely due to the cortical folding being affected only locally where FGF8 was transfected (Fig. 2a, arrows). Because the GI values reflect cortical folding of the entire contour of the brain (Supplementary Fig. S3), it seemed plausible that the GI values were not sensitive enough to detect the changes in local cortical folding. Alternatively, it also seemed possible that newly formed sulci were not deep enough to be detected by the GI values.

The larger GI values in TD ferrets could result from two possible reasons. The first possible reason would be that the number of cortical folds was larger in the TD ferret brain. The second possibility is that the depth of cortical sulci became deeper in the TD ferret brain. To distinguish these possibilities, we newly defined the gyrification number index (GN), which indicates how many times the pial surface is detached from the outer contour of the brain (See the Methods section and Supplementary Fig. S4 for details). Then the GN values from the transfected side of the cortex (GNt) were divided by those from the other side of the cortex (GNo) in the same brain sections. The GNt/GNo values would be 1 if the numbers of cortical folds were same between the transfected side and the other side, and would be larger than 1 if the number of cortical folds was increased on the transfected side. Consistent with the GI values, we found that the GNt/GNo values were significantly larger at P36 (control, 0.94 ± 0.08; TD, 1.43 ± 0.05; Student’s *t*-test; p < 0.01) (Fig. 3d). This finding clearly indicates the number of cortical folds is increased in TD ferrets. Furthermore, in contrast to the GI values, the GNt/GNo values were significantly larger not only at P36 but also at P16 (control, 1.08 ± 0.12; TD, 1.58 ± 0.12; Student’s *t*-test, p < 0.05) (Fig. 3c). The GNt/GNo values seem to be more sensitive to detect abnormalities in cortical folding.

Because it seemed possible that FGF8 electroporated into one side of the brain also affected gyrification on the other side, we examined the GI values of the hemisphere contralateral to the FGF8-electroporated side at P36, and found that they were almost the same as those of the GFP-electroporated control brain (control, 1.55 ± 0.04; TD, 1.59 ± 0.03; Student’s *t*-test, p > 0.05). This result suggests that FGF8 electroporation on one side of the brain does not have apparent effects on gyrification on the other side.

### Histological changes of the cerebral cortex in developing TD ferrets

**Figure 4:**
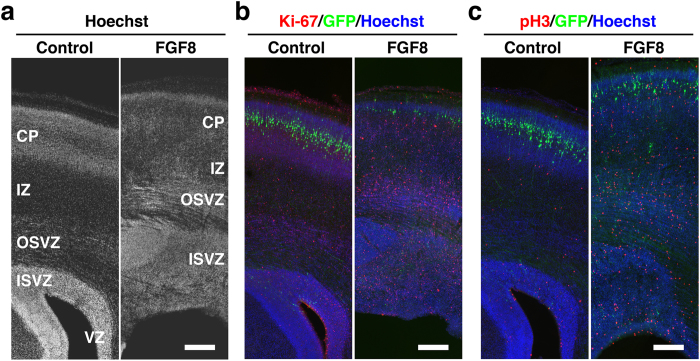
Cell proliferation in the cerebral cortex of developing TD ferrets. GFP and FGF8 were expressed in the ferret cerebral cortex at E33 using *in utero* electroporation, and the brain was prepared at P6. Coronal sections were stained with Hoechst 33342 (white in (**a**), blue in (**b**, **c**)) plus either anti-Ki-67 antibody or anti-phospho-histone H3 antibody (red). Cortical regions containing transfected GFP-positive areas (green) are shown. Note that Ki-67-positive cells and pH3-positive cells were markedly increased in TD ferrets. Scale bars = 300 μm.

We examined histological changes of the cerebral cortex at P6 using coronal sections stained with Hoechst 33342 (Fig. 4a). In the developing cortex of gyrencephalic mammals, there are mainly three types of proliferating cells: radial glial cells (RGs), intermediate progenitor cells (IPs) and outer radial glial cells (oRGs)^30^,^31^,^32^,^33^,^34^,^35^,^36^. RGs (also known as apical progenitors/apical RGs/ventricular RGs) are epithelial stem cells in the ventricular zone (VZ) and generate IPs (also known as basal progenitors) that migrate into the subventricular zone (SVZ). Corticogenesis in higher mammals is distinguished by the appearance of the large SVZ that has an inner (ISVZ) and outer region (OSVZ), often split by a thin layer of fibers called the inner fiber layer (IFL)^37^,^38^. oRGs (also known as OSVZ RGs/basal RGs/intermediate RGs/translocating RGs) are a recently identified novel class of progenitor cells that are abundant in the OSVZ of the developmental cortex in gyrencephalic mammals. Strikingly, we found that the ISVZ and the OSVZ were markedly thicker and contained much more Hoechst-positive cells in the developing cortex of TD ferrets than in control ferrets at P6 (Fig. 4a). Because the ISVZ and the OSVZ contain proliferating cells such as oRGs and IPs, these results raised the possibility that cell proliferation is enhanced in TD ferrets.

To examine if cell proliferation is enhanced in the GFP-positive electroporated cortical area of TD ferrets (Fig. 1a, arrowheads), we performed immunohistochemistry using anti-Ki-67 antibody and anti-phospho-histone H3 (pH3) antibody at P6. We found that the numbers of Ki-67-positive cells and pH3-positive cells were markedly increased in the FGF8-electroporated cortex at P6 (Fig. 4b,c), suggesting that cell proliferation is indeed enhanced in TD ferrets. Many Ki-67-positive cells and pH3-positive cells were predominantly distributed in the ISVZ and the OSVZ, where neural progenitors are dividing. Interestingly, we also found a large number of Ki-67-positive cells and pH3-positive cells in the intermediate zone (IZ) and the cortical plate (CP) of the TD ferret cortex, where dividing cells are rarely found in the normal ferret cortex^39^. This result suggests that cell proliferation is also enhanced in the IZ and the CP.

**Figure 5:**
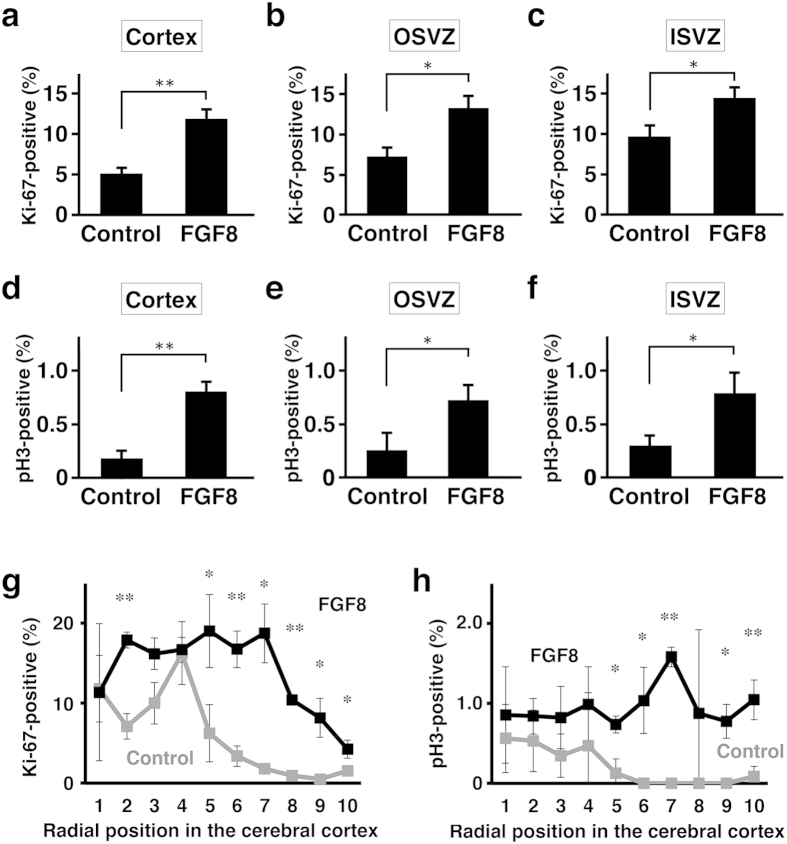
Quantification of cell proliferation in TD ferrets. GFP and FGF8 were expressed in the ferret cerebral cortex at E33 using *in utero* electroporation, and the brain was prepared at P6. Coronal sections containing the anterior part of the lateral ventricle were used for quantification. The numbers of Ki-67-positive cells (**a**–**c**,**g**) and pH3-positive cells (**d**–**f**,**h**) were counted, and were divided by the numbers of Hoechst 33342-positive cells in the same regions. The percentages of positive cells in the cerebral cortex (**a**,**d**), the OSVZ (**b**,**e**) and the ISVZ (**c**,**f**) are shown. (**g**,**h**) The cortex was divided into 10 regions along the radial axis from the ventricular surface (1) to the pial surface (10). The numbers of Ki-67- and pH3-positive cells in each region were counted and were divided by the numbers of Hoechst 33342-positive cells in the same region. The percentages of positive cells are shown. Bars represent mean ± SD. *p < 0.05; **p < 0.01. n = 3 for each condition.

We further examined the percentages of proliferating cells. The numbers of Ki-67-positive cells were counted and divided by those of Hoechst-positive cells. The percentages of Ki-67-positive proliferating cells were more than double in the cerebral cortex of TD ferrets (control, 5.1 ± 0.6%; TD, 11.9 ± 1.1%; Student’s *t*-test, p < 0.01) (Fig. 5a). Interestingly, the percentages of Ki-67-positive cells in TD ferrets were significantly higher in the OSVZ (control, 7.3 ± 1.2%; TD, 13.3 ± 1.5%; Student’s *t-*test, p < 0.05) and in the ISVZ (control, 9.7 ± 1.3%; TD, 14.5 ± 1.3%; Student’s *t*-test, p < 0.05) (Fig. 5b,c). Consistent results were obtained with pH3 staining. The percentages of pH3-positive cells were 4-fold higher in the cerebral cortex of TD ferrets (control, 0.18 ± 0.07%; TD, 0.80 ± 0.09%; Student’s *t*-test, p < 0.01) (Fig. 5d). The percentages of pH3-positive cells in TD ferrets were significantly higher in the OSVZ (control, 0.25 ± 0.16%; TD, 0.72 ± 0.14%; Student’s *t*-test, p < 0.05) and in the ISVZ (control, 0.30 ± 0.09%; TD, 0.79 ± 0.18%; Student’s *t*-test, p < 0.05) (Fig. 5e,f). These results clearly indicate that cell proliferation in the OSVZ and the ISVZ is stimulated.

Because the border between the VZ and the SVZ was often difficult to define in the cerebral cortex of TD ferrets, we also counted Ki-67- and pH3-positive cells using a more objective definition of regions of interest (ROI). We divided the cortex into 10 regions along the radial axis from the ventricular surface to the pial surface (Supplementary Fig. S5), and quantified Ki-67- and pH3-positive cells in each region. The percentages of Ki-67- and pH3-positive cells were significantly larger in the FGF8-transfected cortex than in the GFP-transfected control cortex (Fig. 5g,h), suggesting that cell proliferation is enhanced throughout the cerebral cortex of TD ferrets.

It should be noted that the locations of GFP-positive transfected cells and those whose proliferation had increased were spatially distinct. GFP-positive cells were located in the CP of the cerebral cortex, whereas increased Ki-67- and pH3-positive cells were distributed not only in the CP but also in the IZ and the SVZ (Fig. 4b,c). These results suggest that FGF8 exerts its effects on dividing cells that are in locations distinct from those of GFP-positive somata in the cerebral cortex. FGF8 could diffuse easily in the cortical tissues to exert its effects. Alternatively, FGF8 could be released from GFP-positive axons located close to dividing cells. Consistently, *in situ* hybridization showed that FGF receptors such as *FGFR2* and *FGFR3* were expressed in the VZ and the SVZ of the ferret cerebral cortex at E40 (Supplementary Fig. S6). These expression patterns are consistent with a recent report showing the expression patterns of FGFRs in the ferret cortex at P0^40^. It seems likely that activated FGF receptors in the VZ and the SVZ resulted in the phenotypes of TD ferrets. However, it remains possible that other FGFRs are also involved in the pathogenesis of TD ferrets.

### Neural progenitor cells in the cerebral cortex of TD ferrets

**Figure 6:**
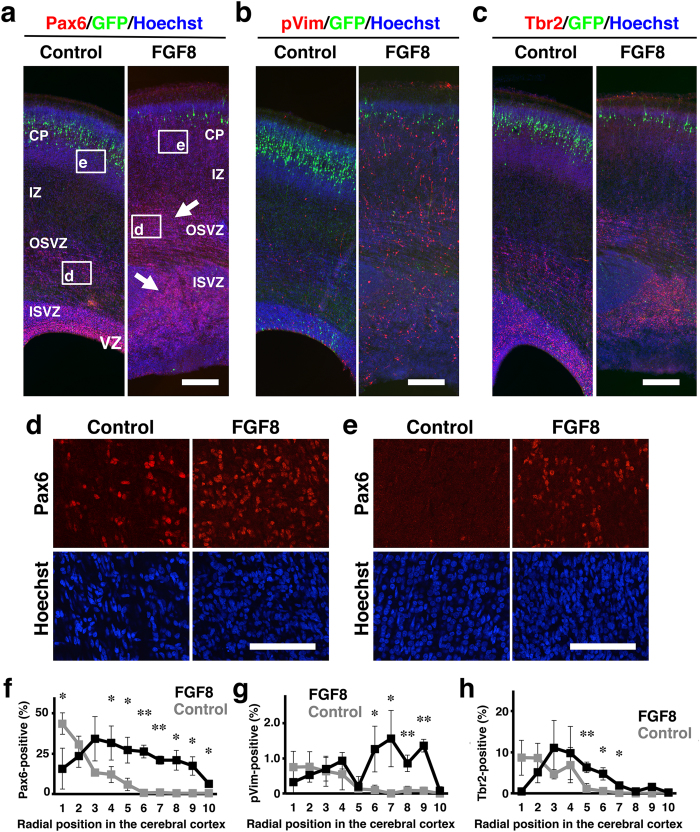
The distribution of Pax6-, pVim- and Tbr2-positive cells in the cerebral cortex of developing TD ferrets. GFP and FGF8 were expressed in the ferret cerebral cortex at E33 using *in utero* electroporation, and the brain was prepared at P6. Coronal sections were stained with Hoechst 33342 (blue) plus either anti-Pax6 antibody, anti-phosphorylated vimentin (pVim) antibody or anti-Tbr2 antibody (red). (**a**–**c**) The cerebral cortex containing the transfected GFP-positive area (green) is shown. Note that Pax6-positive cells (arrows), pVim-positive cells and Tbr2-positive cells were markedly increased in TD ferrets. (**d**) Pax6-positive cells in the OSVZ. Confocal images in the white boxes in (**a**) are shown. (**e**) Pax6-positive cells in the CP. Confocal images in the white boxes in (**a**) are shown. Note that Pax6-positive cells were markedly increased both in the OSVZ and in the CP. (**f**–**h**) The cerebral cortex was divided into 10 regions along the radial axis from the ventricular surface (1) to the pial surface (10). The numbers of Pax6-, pVim- and Tbr2-positive cells in each region were counted and were divided by the numbers of Hoechst 33342-positive cells in the same region. The percentages of positive cells are shown. Bars represent mean ± SD. *p < 0.05; **p < 0.01. n = 3 for each condition. Scale bars = 300 μm (**a**–**c**), 100 μm (**d**,**e**).

Proliferating cells were increased in the ISVZ and the OSVZ of TD ferrets (Fig. 4), raising the possibility that the increased proliferating cells are oRGs and/or IPs. To test this possibility, we examined the expression patterns of Pax6, phosphorylated vimentin (pVim) and Tbr2 at P6. Pax6 and pVim are expressed in RGs and oRGs, and Tbr2 is expressed in IPs. We found that Pax6-positive cells were markedly increased in the ISVZ and the OSVZ of TD ferrets (Fig. 6a, arrows). In addition to an increase in the total number of Pax6-positive cells, their density was also increased in the OSVZ (Fig. 6d). Consistently, pVim-positive cells were increased in the ISVZ and the OSVZ of TD ferrets (Fig. 6b). We also examined the percentages of Pax6- and pVim-positive cells. As described in Supplementary Fig. S5, we quantified the percentages of Pax6- and pVim-positive cells in 10 regions along the radial axis from the ventricular surface to the pial surface. The percentages of Pax6-positive cells and pVim-positive cells were significantly increased in TD ferrets (Fig. 6f,g).

In the TD ferret cortex, a number of Ki-67- and pH3-positive cells were found not only in the ISVZ and the OSVZ but also in the IZ and the CP (Fig. 4), where dividing cells are rarely found in the normal ferret cortex. Consistently, we found that Pax6-positive cells and pVim-positive cells appeared in the IZ and CP of TD ferrets (Fig. 6a,b,e–g), suggesting that oRG-like cells are newly generated in the IZ and CP of TD ferrets. Currently, although the origin of Pax6- and pVim-positive cells in the IZ and the CP is unknown, it seemed possible that oRGs found in the OSVZ of normal ferrets migrated into the IZ and the CP in TD ferrets. Future investigations would be required for uncovering the identities and the roles of oRG-like cells in the IZ and the CP of TD ferrets.

We also examined the distribution patterns of IPs using anti-Tbr2 antibody (Fig. 6c). Tbr2-positive cells were increased in the SVZ of TD ferrets (Fig. 6c), suggesting that the number of IPs is also increased in TD ferrets. Our quantification showed that the percentages of Tbr2-positive cells were also increased in TD ferrets (Fig. 6h). Taken together with the results of Pax6 and pVim, our findings indicate that oRGs and IPs are increased in TD ferrets. It seems likely that these increased oRGs and IPs underlie the pathophysiology of polymicrogyria. On the other hand, we cannot exclude the possibility that displaced RGs are also increased in TD ferrets. The effects of FGF8 on displaced RGs would be an important issue to address in future research.

Recently, it was reported that there are three kinds of gene expression patterns in progenitors: progenitors expressing Pax6 alone, Tbr2 alone and both Pax6 and Tbr2^41^. We therefore performed Pax6 and Tbr2 double immunostaining and counted the numbers of each cell type in the IZ and CP. We found that the percentage of Hoechst 33342-positive cells which were Pax6+/Tbr2- was significantly increased in TD ferrets (control, 1.2 ± 0.3%; TD, 17.6 ± 3.9%; Student’s *t*-test, p < 0.01). The percentages of Pax6+/Tbr2+ cells were also increased in TD ferrets (control, 0.06 ± 0.06%; TD, 1.21 ± 0.43%; Student’s *t*-test, p < 0.05). Pax6-/Tbr2+ cells were comparable between TD and control ferrets (control, 0%; TD, 0.1 ± 0.1%). These results suggest that Pax6-single positive cells and Pax6/Tbr2-double positive cells were increased in TD ferrets. Consistently, we quantified the number of Pax6+/Tbr2-/pH3+ cells in the OSVZ and found that Pax6+/Tbr2-/pH3+ cells were significantly increased in TD ferrets (control, 27.5 ± 4.9 cells/mm^2^; TD, 89.3 ± 19.4 cells/mm^2^; Student’s *t*-test; p < 0.05).

It should be noted that although Pax6-positive cells in the cerebral cortex were markedly increased in TD ferrets, there was a clear decrease in Pax6-positive cells along the ventricular wall (Fig. 6a,f). Consistently, pVim-positive cells tended to be decreased along the ventricular wall in TD ferrets (Fig. 6b,g). These results suggest that RGs were reduced in the VZ of TD ferrets. Currently, the reasons for this reduction of RGs in the VZ are unclear, but it seems possible that FGF8 induces differentiation of RGs into IPs and oRGs and/or translocation of RGs into the cerebral cortex.

### Astrocytes and oligodendrocytes in the cerebral cortex of TD ferrets

**Figure 7:**
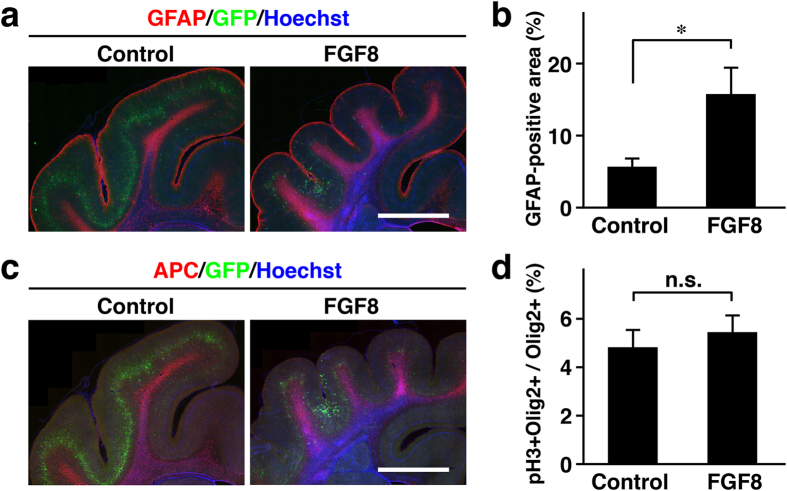
Astrocytes and oligodendrocytes in the cerebral cortex of TD ferrets. GFP and FGF8 were expressed in the ferret cerebral cortex at E33 using *in utero* electroporation, and the brain was prepared at P6 (**d**) and P36 (**a–c**). Coronal sections were subjected to immunohistochemistry. (**a**) Sections were stained with anti-GFAP antibody (red) and Hoechst 33342 (blue). The part of the cerebral cortex containing the transfected GFP-positive area (green) is shown. (**b**) Quantification of the GFAP-positive area in the cortex. The percentage of the total area which was GFP-positive is shown. (**c**) Sections were stained with anti-APC antibody (red) and Hoechst 33342 (blue). The part of the cerebral cortex containing the transfected GFP-positive area (green) is shown. (**d**) The percentage of pH3- and Olig2-double positive cells in Olig2-positive cells. Bars represent mean ± SD. *p < 0.05; n.s., not significant. n = 3 for each condition. Scale bars = 3 mm.

We next examined the expression patterns of GFAP and APC, markers for astrocytes and oligodendrocytes, respectively (Fig. 7). Even in the cortical area showing polymicrogyria, the distribution patterns of GFAP were similar to those in the normal cortex (Fig. 7a). GFAP-positive cells were preferentially located in the cortical surface and the area just under the cortical plates (Fig. 7a). Interestingly, when we quantified the area containing GFAP-positive signals in the cerebral cortex (Supplementary Fig. S7), we found that the GFAP-positive area was significantly larger in TD ferrets (control, 5.6 ± 0.78%; TD, 15.8 ± 3.4%; Welch’s *t*-test; p  <  0.05) (Fig. 7b). These results suggest that astrocytes were increased in TD ferrets. It would be intriguing to examine if increased astrocytes are responsible for making additional gyri.

The distribution of oligodendrocytes was revealed with APC staining (Fig. 7c). The distribution patterns of APC were not apparently affected in TD ferrets. APC was similarly distributed in the white matter in both control and TD ferrets (Fig. 7c). Olig2 is generally considered to be a marker of the oligodendrocyte lineage, although it was reported that Olig2 is also expressed in putative astrocyte precursors in neonatal rodent brains^42^. Quantification of pH3- and Olig2-double positive cells in Olig2-positive cells did not show significant differences at P6 (Fig. 7d), suggesting that proliferation of Olig2-positive cells is not enhanced in TD ferrets. These results suggest that oligodendrocytes are not affected in TD ferrets.

### Layer structures of the cerebral cortex of TD ferrets

**Figure 8:**
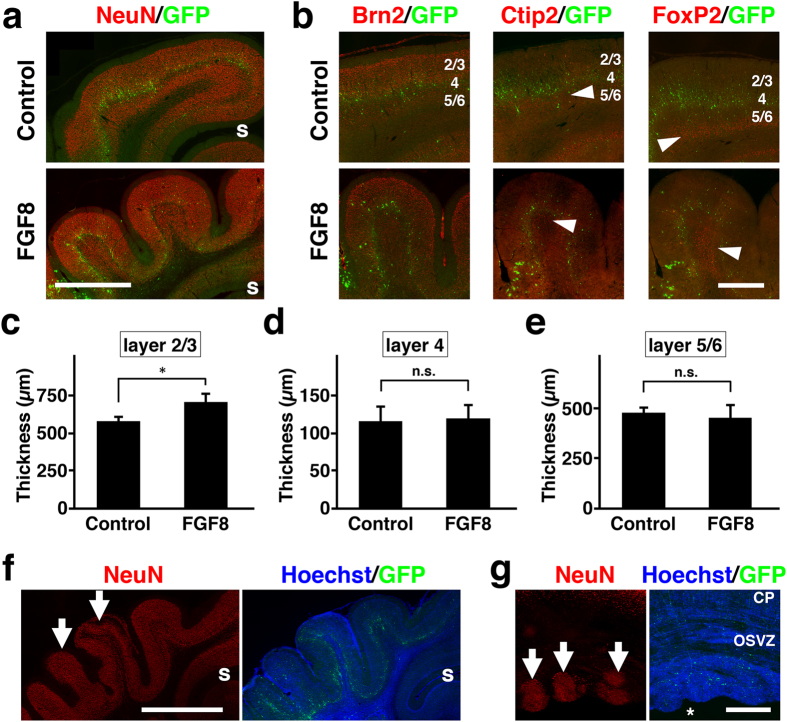
Layer structures in the cerebral cortex of TD ferrets. GFP and FGF8 were expressed in the ferret cerebral cortex at E33 using *in utero* electroporation, and the brain was prepared at P6 (**g**) and P36 (**a**–**f**). Using coronal sections, immunostaining (red) and Hoechst 33342 staining (blue) were performed. GFP-positive transfected cells are shown in green. (**a**) NeuN immunostaining. Lower magnification images of the cerebral cortex of control ferrets and polymicrogyria of TD ferrets are shown. s, splenial sulcus. (**b**) Higher magnification images of polymicrogyria with normal layer structures. Layer-specific expression patterns of Brn2, Ctip2 and FoxP2 were preserved in the cortical area with polymicrogyria. Arrowheads indicate immunopositive cells. Numbers indicate layers in the cortex. (**c–e**) The average thicknesses of layer 2/3 (**c**), layer 4 (**d**) and layer 5/6 (**e**). Note that layer 2/3 was significantly thicker in TD ferrets than in control ferrets. Bars represent mean ± SD. *p < 0.05; n.s., not significant. (**f**) Lower magnification images of polymicrogyria with distorted layer structures. Layer 2/3 neurons were predominantly increased (arrows). s, splenial sulcus. (**g**) Subependymal heterotopia (arrows). CP, cortical plate; OSVZ, outer subventricular zone. Asterisk indicates the lateral ventricle. Scale bars = 2 mm (**a**), 1 mm (**b**), 3 mm (**f**) and 500 μm (**g**).

To examine the cortical layer structures of polymicrogyria in TD ferrets, we used anti-NeuN antibody that recognizes post-mitotic neurons. At P36, when polymicrogyria became apparent (Fig. 1), we found two kinds of cortical structures. First, we found polymicrogyria with almost normal cortical layer structures (Fig. 8a). Interestingly, overall cortical structure revealed with NeuN immunostaining seemed to be preserved. On the other hand, even though the cytoarchitectonic structures of the cerebral cortex seemed normal in TD ferrets, it remained possible that neuronal identities were affected. We therefore examined the layer markers such as Brn2, Ctip2 and FoxP2. As in the case of mice^43^,^44^,^45^, Brn2 was distributed in layers 2/3/5 in the control ferret cortex at P36 (Fig. 8b, control). Ctip2 was predominantly expressed in layer 5 but also in layer 6, whereas FoxP2 was mainly distributed in layer 6 but also in layer 5 (Fig. 8b, control). Consistent with our observation using NeuN staining, Brn2 immunostaining showed normal 6-layered structures in the cortical area with polymicrogyria in TD ferrets (Fig. 8b). Ctip2 and FoxP2 immunostaining also showed that the cortical area with polymicrogyria in TD ferrets contained apparently normal layers 5 and 6 (Fig. 8b, arrowheads). These results suggest that neuronal identities are not affected in TD ferrets. Although the cortical layer structures of polymicrogyria in TD ferrets were preserved, it seemed possible that the thicknesses of cortical layers were affected differently in TD ferrets. To test this possibility, we measured the average thicknesses of layers 2/3, 4 and 5/6 in coronal sections at P36. Interestingly, we found that layer 2/3 was significantly thicker in TD ferrets (control, 582 ± 23 μm; TD, 708 ± 52 μm; Student’s *t*-test; p < 0.05) (Fig. 8c). In contrast, the thicknesses of layer 4 and layer 5/6 were not increased in TD ferrets (Fig. 8d,e). These results suggest that layer 2/3 was preferentially increased compared with other layers in TD ferrets. The increase in layer 2/3 could be involved in the formation of polymicrogyria in TD ferrets.

Second, we also found polymicrogyria in which layer 2/3 was predominantly distorted, but in which other layers seemed less affected (Fig. 8f, arrows). Overproduction of layer 2/3 neurons could result from enhanced cell proliferation at the later stage of cortical development. Beside polymicrogyria, it has been reported that human TD patients also have subependymal heterotopia^8^, which is the presence of heterotopic nodules situated close to the lateral ventricle. Consistently, we found heterotopic nodules close to the lateral ventricle in the cerebral cortex of TD ferrets (Fig. 8g, arrows). Interestingly, the subependymal heterotopia in TD ferrets was NeuN-positive, suggesting that subependymal heterotopia contains many post-mitotic neurons.

## Discussion

We have shown that FGF8-transfected ferrets have polymicrogyria, megalencephaly and subependymal heterotopia, which are common features of human TD patients. Our histological analyses indicate that oRGs and IPs are increased in the SVZ of TD ferrets. Polymicrogyria seemed to result from increased numbers of oRGs and IPs. Our findings also suggest that TD ferrets have increased astrocytes and thickening in layer 2/3, which could be involved in the pathogenesis of polymicrogyria.

### Pathophysiology of the cortical malformation of TD

Because embryonic and neonatal samples of human TD patients are often difficult to obtain, the mechanisms underlying of the cortical malformation of TD are still largely unclear. Because embryonic and neonatal ferret samples are available, TD ferrets enabled us to investigate the pathophysiological mechanisms of TD. In contrast to TD mice, our TD ferrets exhibited polymicrogyria and subependymal heterotopias, as in the case of human TD patients^8^,^9^. We found that cell proliferation was significantly enhanced, and oRGs and IPs were increased in neonatal TD ferrets. A recent work reported that progenitor cells were increased in the cortex in human TD, but detailed information about the identities of progenitor cells were unclear^46^. A previous pioneering work clearly demonstrated that IPs were increased in TD mice^47^. In addition to IPs, we uncovered that oRGs are increased in TD ferrets. Therefore, it would be intriguing to examine the expression of Pax6 and pVim in human TD patients. In addition, we found oRG-like cells in the IZ and the CP of TD ferrets. The origin and the role of these cells in the pathophysiology of TD would be interesting to examine.

Our findings showed that astrocytes and the thickness of layer 2/3 were preferentially increased in TD ferrets. It seems likely that increased oRGs and IPs result in the overproduction of layer 2/3 neurons and astrocytes. Previously, it has been hypothesized that the appearance of cortical gyri was produced by an increase in superficial layers relative to deep layers of the cerebral cortex^31^,^48^. Our finding is consistent with this hypothesis. In addition, our findings raise the possibility that the increase in astrocytes is involved in the formation of cortical gyri. Although the amount of astrocytes compared to that of neurons in the cortex gradually increased with evolution^49^, little attention has been paid to the role of astrocytes in the production of cortical gyri. It would be extremely important to examine the roles of oRGs, IPs, upper cortical neurons and astrocytes in the formation of cortical gyri.

There have been two hypotheses about the pathophysiology of TD^8^. It has been suggested that activated FGF signaling in the brain leads to the brain phenotype of TD. Alternatively, it was also proposed that the brain phenotype of TD is a secondary deformation caused by skull defects such as craniosynostosis. A previous report using mice showed that genetic manipulations that introduce a TD mutation only in the nervous system led to megalencephaly, suggesting that megalencephaly results from activated FGF signaling in the brain^19^. In contrast to megalencephaly, because polymicrogyria was not obvious in TD mice^18^,^19^, it was difficult to address which hypotheses accounted for the pathophysiology of polymicrogyria using TD mice. Because we expressed FGF8 only in the nervous system, our study using TD ferrets directly indicate that as in the case of megalencephaly, FGF signaling in the brain is responsible for polymicrogyria. Consistently, the gyral abnormalities in human TD patients become evident before synostosis occurs during development.

### Activation of FGF signaling produces polymicrogyria in ferrets

We found that activation of FGF signaling results in polymicrogyria in ferrets. Because we also found that oRGs and IPs were markedly increased in the developing cortex of TD ferrets, one attractive hypothesis would be that increased oRGs are responsible for polymicrogyria of TD ferrets. Consistently, it has been proposed that increased number of oRGs led to the emergence of gyrencephalic cortex during evolution^30^,^31^,^34^,^35^. Because it has been demonstrated that activation of FGF signaling increases cell proliferation of progenitor cells in the developing cortex^50^,^51^,^52^,^53^,^54^, it seems plausible that activation of FGF signaling leads to proliferation of oRGs.

Instead of using FGF8, it seemed possible to express a constitutively active form of FGFR3 (caFGFR3) in the ferret brain using *in utero* electroporation to make TD ferrets. However, we chose FGF8 because of the following reason. Using *in utero* electroporation, the number of transfected neurons is limited. For example, if *in utero* electroporation is performed at E31, lower cortical neurons rather than upper cortical neurons are preferentially transfected^25^. Therefore, FGF signaling seemed to be activated only in a subset of neurons if caFGFR3 is expressed using *in utero* electroporation because caFGFR3 works cell-autonomously. In order to activate FGF signaling in a large number of neurons, it was desirable to use a secreted molecule such as FGF8 rather than a trans-membrane molecule like caFGFR3.

### Advantages of ferrets for examining the mechanisms of TD

Previously, it was reported that TD mice showed megalencephaly, but failed to exhibit polymicrogyria^18^,^19^. In contrast, our TD ferrets have obvious polymicrogyria. Currently, the reasons for this discrepancy between ferrets and mice are unclear, but it seems plausible that mice do not have the mechanisms responsible for making polymicrogyria because the cortical gyrus is missing in normal mice. Ferrets seem to be more appropriate for investigating the pathophysiology of cortical malformations.

Ferrets have several advantages. First, the brain of ferrets has been widely used for anatomical and electrophysiological experiments, so anatomical and electrophysiological data are available. The anatomical structures of cortical gyri and sulci are well described^28^. Second, the developmental processes of cortical gyri have been revealed^28^. Notably, when ferret babies are born, their brains are lissencephalic, and cortical gyri and sulci are formed after birth. Therefore, the mechanisms of the formation and malformation of cortical gyri can be examined using neonatal ferrets rather than embryos. Third, usually more than 6 ferret babies are born from one pregnant mother. This large number of babies per pregnant mother enables us to examine various experimental conditions and to obtain a sufficient number of experimental samples.

In addition to polymicrogyria, ferrets might be also useful for investigating the pathophysiology of cortical malformations such as lissencephaly and pachygyria and neurological disorders like schizophrenia and autism. It would be intriguing to investigate behavioral phenotypes in ferret models of cortical malformation. As exemplified in this study, genetic manipulations of ferrets using *in utero* electroporation should open the door to the next generation of neuroscience experiments using gyrencephalic higher mammals.

## Methods

### Animals

Normally pigmented, sable ferrets (*Mustela putorius furo*) were purchased from Marshall Farms (North Rose, NY). Ferrets were maintained as described previously^20^,^21^,^22^. The day of birth was counted as postnatal day 0 (P0). All procedures were performed in accordance with protocols approved by the Animal Care Committee of Kanazawa University. Experiments were repeated at least three times and gave consistent results.

### *In utero* electroporation for ferrets

*In utero* electroporation using ferrets was performed as described previously^24^,^25^. Briefly, pregnant ferrets were deeply anesthetized, and their body temperature was maintained using a heating pad. The uterine horns were exposed and kept wet by adding drops of phosphate-buffered saline (PBS) intermittently. The location of embryos was visualized with transmitted light delivered through an optical fiber cable. The pigmented iris was visible, and this enabled us to assume the location of the lateral ventricle. Approximately 2–5 μl of DNA solution was injected into the lateral ventricle at the indicated ages using a pulled glass micropipette. Each embryo within the uterus was placed between tweezer-type electrodes with a diameter of 5 mm (CUY650-P5, NEPA Gene, Japan). Square electric pulses (100 V, 50 ms) were passed 5 times at 1-second intervals using an electroporator (ECM830, Harvard Apparatus, MA). The wall and skin of the abdominal cavity were sutured, and the embryos were allowed to develop normally.

### Plasmids

pCAG-GFP was described previously^55^. To make pCAG-FGF8, the GFP of pCAG-GFP was replaced with mouse FGF8b. Plasmids were purified using an Endofree Plasmid Maxi kit (Qiagen, Germany). Prior to *in utero* electroporation experiments, plasmid DNA was diluted to 1–3 mg/ml in PBS, and Fast Green solution was added at a final concentration of 0.5% to monitor the injection.

### Immunohistochemistry

Immunohistochemistry was performed as described previously with slight modifications^56^,^57^,^58^. Briefly, ferrets were deeply anesthetized and transcardially perfused with 4% paraformaldehyde (PFA)/PBS. After the brain was dissected, the brain was cryoprotected by being immersed in 30% sucrose for 3 days and embedded in OCT compound.

Coronal sections (50 μm) were made using a cryostat. Sections containing the anterior part of the lateral ventricle were used for further analyses. The sections were permeabilized with 0.5% Triton X-100/PBS and incubated overnight with primary antibodies, which included anti-Ki-67 antibody (Leica), anti-phospho-histone H3 (pH3) antibody (Upstate Biotechnology), anti-pH3 antibody (Millipore), biotin-conjugated anti-pH3 antibody (Millipore), anti-Olig2 antibody (Immuno-Biological Laboratories, Japan), anti-Pax6 antibody (Abcam), anti-Pax6 antibody (Covance), anti-phosphorylated vimentin (pVim) antibody (Medical & Biological Laboratories, Japan), anti-Tbr2 antibody (Abcam), anti-NeuN antibody (Chemicon), anti-GFAP antibody (Sigma-Aldrich), anti-APC antibody (Calbiochem), anti-Brn2 antibody (Santa Cruz Biotechnology), anti-Ctip2 antibody (Abcam) and anti-FOXP2 antibody (Abcam). After being incubated with secondary antibodies and Hoechst 33342, the sections were washed and mounted. Experiments were repeated at least three times and gave consistent results.

### *In situ* hybridization

Sections prepared from fresh-frozen tissues or fixed tissues were treated with 4% paraformaldehyde for 10 min, 1 μg/ml proteinase K for 10 min and 0.25% acetic anhydride for 10 min. After prehybridization, the sections were incubated overnight at 58 °C with digoxigenin-labeled RNA probes diluted in hybridization buffer (50% formamide, 5× SSC, 5× Denhardt’s solution, 0.3 mg/ml yeast RNA, 0.1 mg/ml herring sperm DNA, and 1 mM DTT). The sections were then incubated with alkaline phosphatase-conjugated anti-digoxigenin antibody (Roche, Indianapolis, IN) and were visualized using NBT/BCIP as substrates. Experiments were repeated at least three times in different animals and gave consistent results.

### Microscopy

Epifluorescence microscopy was performed with an AxioImager A1 microscope (Carl Zeiss) or a BIOREVO BZ-9000 (Keyence). Confocal microscopy was performed with an FV10i FLUOVIEW microscope (OLYMPUS).

### Cell counting

Confocal microscopic images were acquired using an FV10i FLUOVIEW microscope (OLYMPUS). The numbers of Hoechst 33342-positive cells were counted automatically using Metamorph software. The numbers of immunopositive cells were manually counted using the “cell counter” function of ImageJ software, and were divided by the numbers of Hoechst 33342-positive cells.

### Quantification of cortical folding

Serial coronal sections with 50 μm thickness were prepared, and sections containing the anterior part of the lateral ventricle were used for further analyses. After staining with Hoechst 33342, images were acquired using a BZ-9000 microscope (Keyence).

The gyrification index (GI) was calculated as described previously (Supplementary Fig. S3)^29^. Briefly, the length of the complete contour and that of the outer contour of the electroporated side of the cortical hemisphere were measured. The ratio of the lengths of these two contours was calculated as follows.

The GI value = the length of the complete contour/the length of the outer contour

We newly defined the gyrification number index (GN), which indicates how many times the pial surface was detached from the outer contour of the brain (Supplementary Fig. S4). Then the GN values of the transfected side (GNt) were divided by those of the other control side (GNo). The resultant GNt/GNo values would be 1 if the numbers of cortical folds were same between the manipulated side and the control side of the cerebral cortex. The GNt/GNo values would be larger than 1 if the numbers of cortical folds in the manipulated side were larger than those in the control side.

### Quantification of the sizes of the brain and the lateral ventricle

Coronal sections containing the anterior part of the lateral ventricle were used for quantification. The area within the complete contour (see Supplementary Fig. S3, green line) of either the transfected side or the other side of the brain was measured using ImageJ software. To minimize the variation of the size depending on the position of coronal sections, we calculated the ratio of the size of the transfected side and that of the other side. The ratio would be 1 if the size of the transfected side was same as that of the other side, and would be larger than 1 if the size of the transfected side was larger than that of the other side. Similarly, the size of the lateral ventricle was measured. The area of the lateral ventricle of either the transfected side or the other side of the brain was measured using ImageJ software, and the ratio of the size of the transfected side and that of the other side was calculated.

### Quantification of the average thickness of cortical layers

Coronal sections containing the anterior part of the lateral ventricle obtained at P36 were used for quantification. To calculate the average thickness of layers 2/3, 4 and 5/6 around the coronal sulcus, the area of each layer was measured over a length of at least 5 mm and was subsequently divided by the length of each layer. Layers were identified using Brn2, Ctip2 and FoxP2 immunostaining and Hoechst 33342 staining.

### Quantification of the GFAP-positive area

Coronal sections containing the anterior part of the lateral ventricle obtained at P36 were stained with anti-GFAP antibody and Hoechst 33342, and were used for quantification (Supplementary Fig. S7). The region of the cortical hemisphere located dorsal to the corpus callosum was selected, and the selection excluded the cortical surface (Supplementary Fig. S7, broken line). The area within the broken line and the area containing GFAP signals within the broken line were measured using ImageJ software, and the latter was divided by the former.

### Statistical analyses

Statistical analyses were performed using Statcel3 software (OMS Publishing, Japan). After the *F*-test was performed, the *p* values were determined using a Student’s *t*-test or a Welch’s *t*-test. “n” means the number of animals.

## Additional Information

**How to cite this article**: Masuda, K. *et al*. Pathophysiological analyses of cortical malformation using gyrencephalic mammals. *Sci. Rep.* **5**, 15370; doi: 10.1038/srep15370 (2015).

## Supplementary Material

Supplementary Information
