# Supplementary material for: Pathophysiological analyses of cortical malformation using gyrencephalic mammals
Source: Sci Rep. 2015 Oct 20;5:15370. doi: 10.1038/srep15370 (PMC4613358; doi:10.1038/srep15370)
Supplement: Supplementary Information [file srep15370-s1.pdf]

Supplementary Information

Pathophysiological analyses of cortical malformation using gyrencephalic mammals

Kosuke Masuda, Tomohisa Toda, Yohei Shinmyo, Haruka Ebisu, Yoshio Hoshiba,

Mayu Wakimoto, Yoshie Ichikawa and Hiroshi Kawasaki

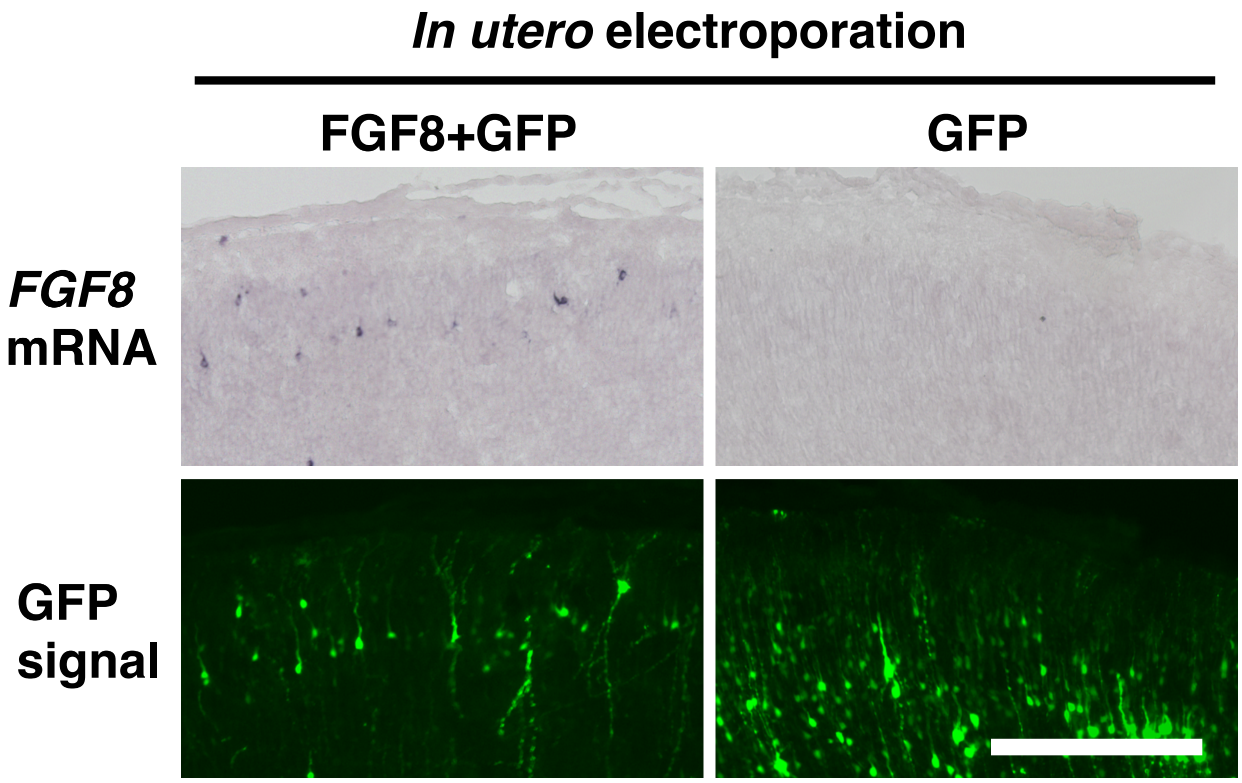

Supplementary Figure S1. The distribution patterns of ectopically expressed mouse *FGF8* in the ferret cerebral cortex. After *in utero* electroporation was performed at E33, brains were prepared at P6, and coronal sections of the cerebral cortex were subjected to *in situ* hybridization with a mouse FGF8 probe. Note that mouse *FGF8* mRNA was detected in GFP-positive cells of the FGF8-transfected cortex, whereas no signals were detected in the control GFP-transfected cortex. Scale bar = 300 μm.

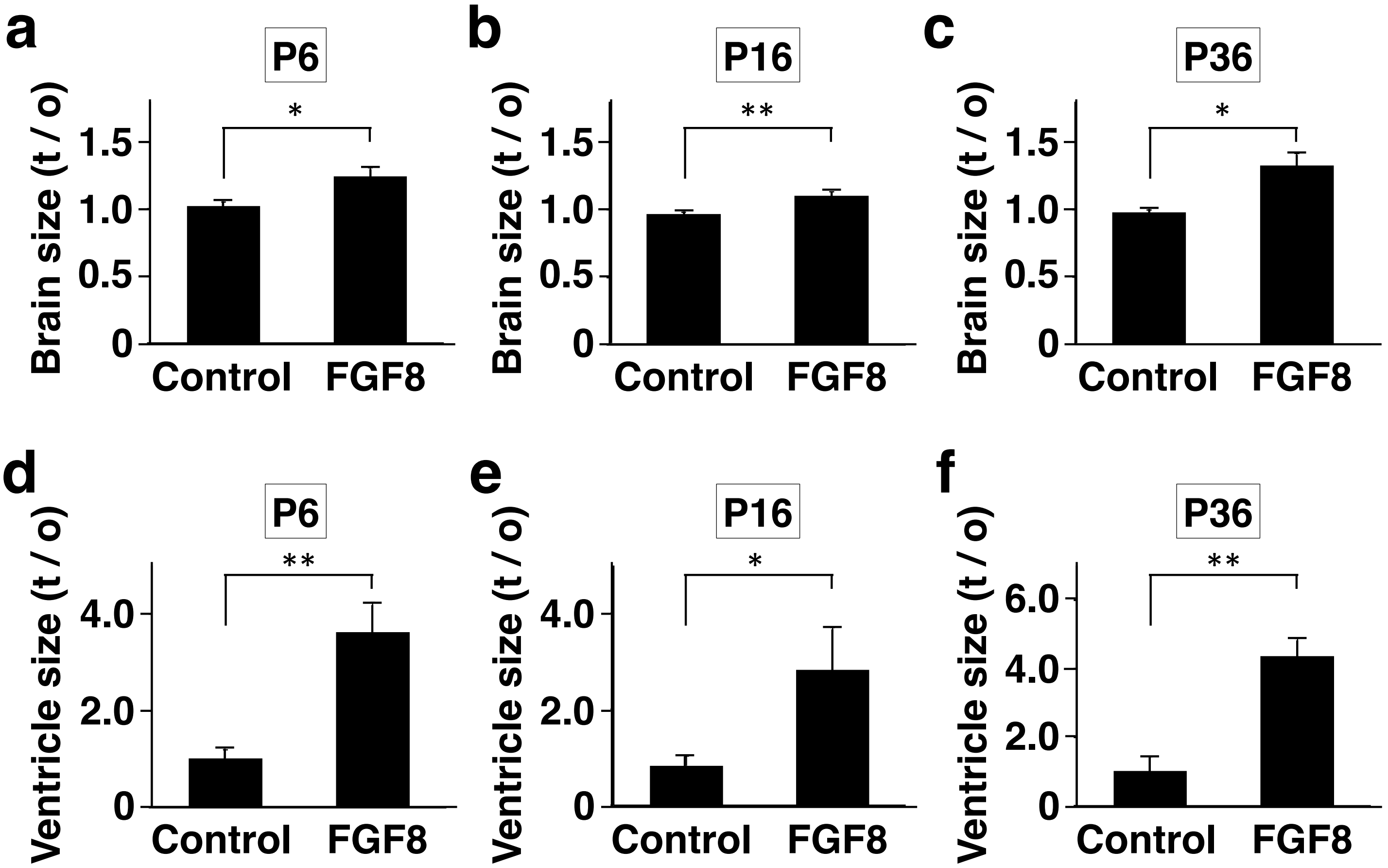

Supplementary Figure S2. Quantification of the sizes of the brain and the lateral ventricle. Coronal sections containing the anterior part of the lateral ventricle were used for quantification. To minimize the variation of the size depending on the position of coronal sections, we calculated the ratio of the transfected side (t) and the other side (o). The t/o ratio would be 1 if the size of the transfected side was same as that of the other side, and would be larger than1 if the size of the transfected side was larger than that of the other side. (**a-c**) The brain size was significantly increased in the FGF8-transfected hemisphere than in the GFP-transfected control hemisphere. The area of the transfected hemisphere and that of the other side of the hemisphere were measured, and the ratio of these two values are shown. (**d-f**) The sizes of lateral ventricle were significantly increased in the FGF8-transfected hemisphere compared to the GFP-transfected control hemisphere. The area of the lateral ventricle of the transfected side and that of the other side were measured, and the ratio of these values are shown. Bars represent mean ± SD. *, $p<0.05$; **, $p<0.01$. n=3 for each condition.

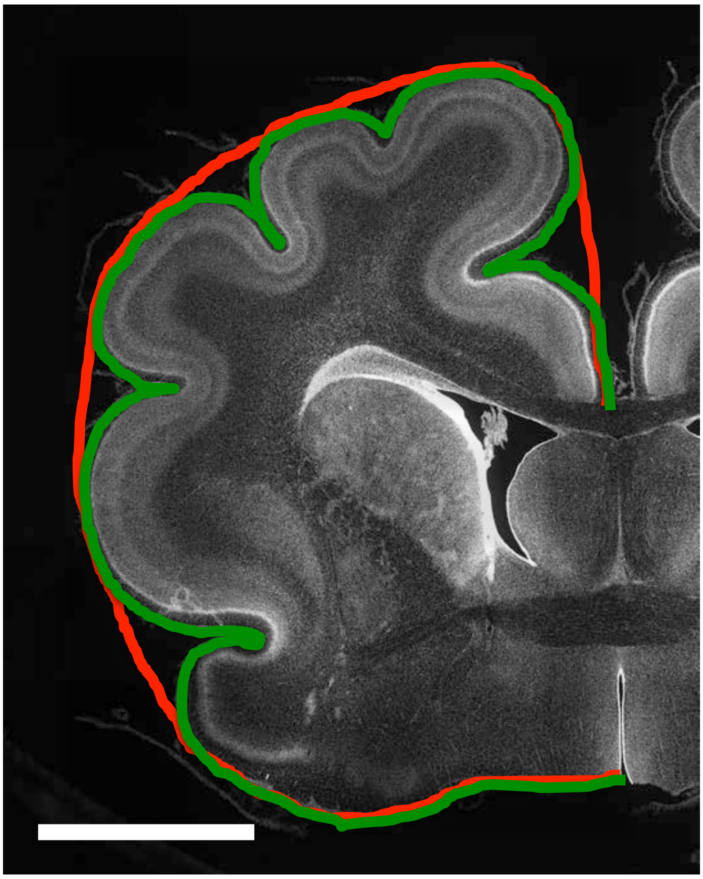

$$\text{The GI value} = \frac{\text{Length of the complete contour}}{\text{Length of the outer contour}}$$

Supplementary Figure S3. The definition of the GI value. After *in utero* electroporation was performed, brains were prepared at P16 and P36. Coronal sections containing the anterior part of the lateral ventricle were stained with Hoechst 33342 (white). The GI value was the length of the complete contour (green line) divided by that of the outer contour (red line). Scale bar = 6 mm.

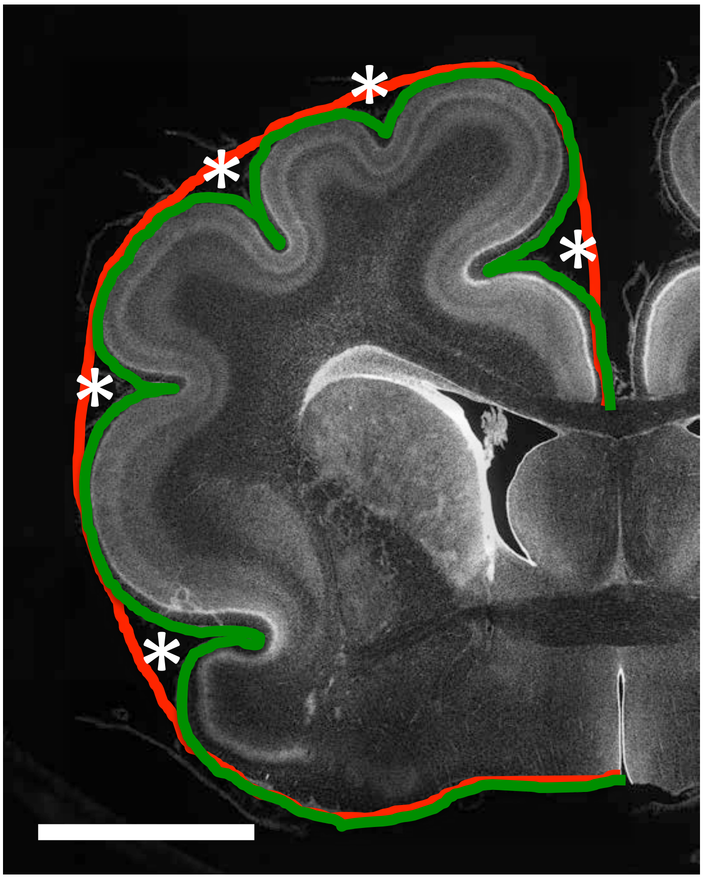

The GN value = the number of asterisks

The GNt value = the GN of transfected side.
The GNo value = the GN of the other side.

Supplementary Figure S4. The definition of the GN value. After *in utero* electroporation was performed, brains were prepared at P16 and P36. Coronal sections containing the anterior part of the lateral ventricle were stained with Hoechst 33342 (white). The GN value indicates how many times the complete contour (green line) was detached from the outer contour (red line) of the brain (i.e. the number of asterisks). Scale bar = 6 mm.

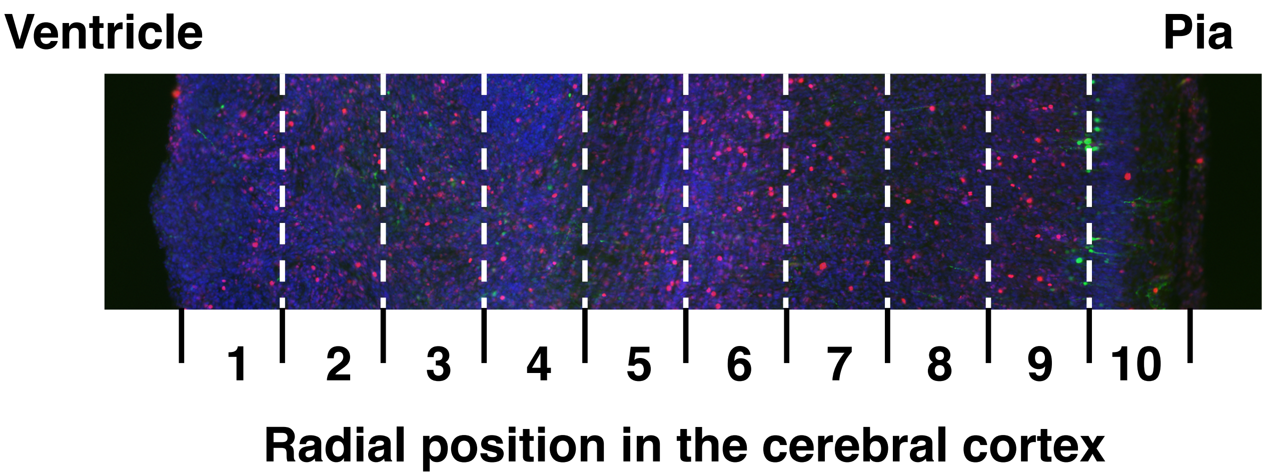

Supplementary Figure S5. Quantification of positive cells in 10 divided regions in the cerebral cortex. Images of the cerebral cortex were divided into 10 regions along the radial axis from the ventricular surface (1) to the pial surface (10). The number of positive cells (red) in each region was counted and divided by that of Hoechst 33342-positive cells (blue) in the same region.

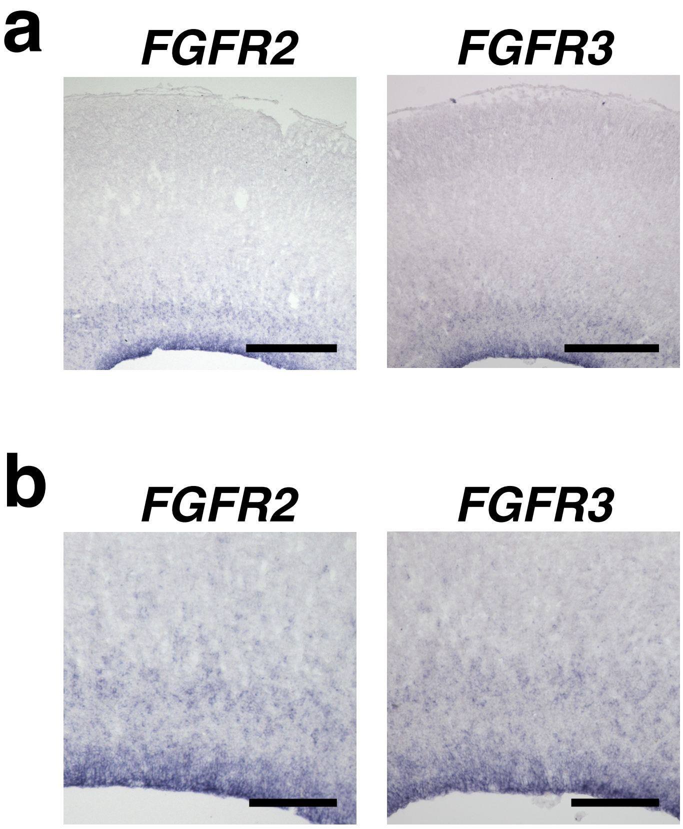

Supplementary Figure S6. Expression patterns of FGF receptors in the developing ferret cortex. Expression patterns of ferret *FGFR2* and *FGFR3* mRNA in coronal sections of the ferret cerebral cortex were examined using *in situ* hybridization at E40. Note that FGF receptors were mainly expressed in the VZ and the SVZ. (**a**) Low magnification images of the cerebral cortex. (**b**) High magnification images of the VZ and the SVZ. Scale bars = 500 μm (**a**), 200 μm (**b**).

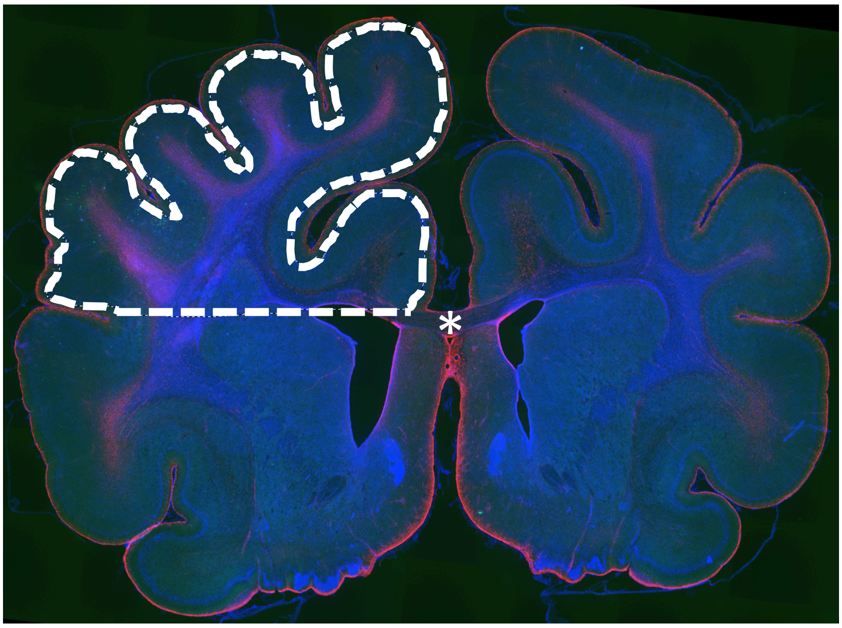

Supplementary Figure S7. Quantification of the GFAP-positive area in the cerebral cortex. After *in utero* electroporation was performed, brains were prepared at P36. Coronal sections containing the anterior part of the lateral ventricle were stained with anti-GFAP antibody (red) and Hoechst 33342 (blue). The region of the cortical hemisphere located dorsal to the corpus callosum (asterisk) was selected, and the selection excluded the cortical surface. The total area (area within the broken line) and the GFAP-positive area (red area within the broken line) were measured. Then, the value of the GFAP-positive area was divided by that of the total area.

CP, cortical plate
FGF, fibroblast growth factor
FGFR, fibroblast growth factor receptor
GI, gyrification index
GN, gyrification number index
GNo, the GN values from the other control side
GNt, the GN values from the transfected side
IFL, inner fiber layer
IPs, intermediate progenitor cells
ISVZ, inner SVZ
IZ, intermediate zone
oRGs, outer radial glial cells
OSVZ, outer SVZ
pH3, phospho-histone H3
pVim, phosphorylated vimentin
RGs, radial glial cells
SVZ, subventricular zone
TD, thanatophoric dysplasia
VZ, ventricular zone

Supplementary Table. Abbreviations used in this study.
